# Association between Satisfaction with Life and Personality Types A and D in Young Women with Acne Vulgaris

**DOI:** 10.3390/ijerph17228524

**Published:** 2020-11-17

**Authors:** Karolina Chilicka, Aleksandra M. Rogowska, Renata Szyguła, Ewa Adamczyk

**Affiliations:** 1 Institute of Health Sciences, University of Opole, 45-040 Opole, Poland; renata.szygula@uni.opole.pl (R.S.); ewa.adamczyk@uni.opole.pl (E.A.); 2 Institute of Psychology, University of Opole, 45-052 Opole, Poland; arogowska@uni.opole.pl

**Keywords:** acne vulgaris, Framingham Type A Scale, DS14, satisfaction with life, SWLS, Type D personality, Type A Behavior Pattern, women

## Abstract

People with acne vulgaris report a lower level of satisfaction with life and are more frequently classified as having Type D personalities than those without acne. This research examined, for the first time, the moderating and mediating role of personality type in the relationship between acne severity and satisfaction with life. Among 300 female nursing and cosmetology students ranging in age from 19 to 24 years (M = 21.28, SD = 1.39), 150 individuals (50%) presented with symptoms of acne vulgaris (AV group), while the other 150 (50%) were categorized as controls without acne vulgaris (WAV sample). A cross-sectional study was conducted using three self-report questionnaires: The Satisfaction with Life Scale (SWLS), the Framingham Type A Scale (FTAS), and the Type D Scale (DS14). Acne vulgaris was clinically diagnosed using the Hellgren–Vincent Scale (HVS). The AV group scored significantly higher on the FTAS and DS14 and lower on the SWLS than the WAV sample. Life satisfaction correlated negatively with both the negative affectivity (NA) and social inhibition (SI) subscales of the DS14. The moderating role of the Type A behavioral pattern (TABP) and the mediating role of both NA and SI subscales of the DS14 were observed in the relationship between acne severity and satisfaction with life. The type of personality may explain the mechanism of the relationship between acne disease and subjective well-being. Therefore, psychological interventions and strategies focused on managing stress and mood may effectively improve satisfaction with life in people with acne.

## 1. Introduction

Acne is a chronic inflammatory disease of the sebaceous unit, which occurs most frequently in adolescence or early adulthood, between 15 and 24. Acne persists from adolescence into adulthood in around 64% among people in their twenties and 43% of individuals in their thirties [1]. This disease’s characteristic skin eruptions are comedones, pustules, papules, cystic nodules, and inflammatory or noninflammatory lesions, leading to visible scars. In women, skin lesions appear most often on the face, which may significantly impact patients’ well-being [2,3,4,5]. Among female medical students, approximately 61% complained that their acne treatment was a problem, and almost 90% reported that acne impacts their psychosocial functioning [6].

People struggling with acne disease often feel isolated, excluded from society, and usually limit their social life [7,8]. Among the key areas of health-related quality of life affected by acne, Fabbrocini et al. [9] identified emotional functioning, social functioning, relationships, leisure activities, daily activities, sleep, and school or work. Furthermore, a study on the psychological burden of adult acne patients from 13 European countries (including Poland) showed that people with acne had higher depression and anxiety scores than their control counterparts [10]. A review of such studies evidenced that acne vulgaris impacts psychosocial health in terms of depression, suicidal ideation, anxiety, psychosomatic symptoms (including pain and discomfort), embarrassment, and social inhibition [11,12].

It has been shown that the level of quality of life is significantly lower in people suffering from acne than those without this dermatologic disease [13,14]. Moreover, research indicates that acne severity may have a considerable adverse impact on quality of life and self-esteem [15,16,17]. People with moderate and severe acne report more significant psychosocial and emotional impairment, greater risk of anxiety and depression, and lower quality of life than those with mild symptoms [18,19,20].

Quality of life is dependent on subjective well-being (SWB), which refers to all kinds of evaluations that people perform about their own lives. SWB includes emotional states (both positive and negative affect) and life satisfaction (happiness), understood as a cognitive–judgmental aspect of well-being [21]. Deiner et al. [22] developed the Satisfaction with Life Scale (SWLS) to measure the global cognitive self-judgment of an individual’s entire life by using the person’s individual criteria across a broad set of human activities [23]. An individual can rate on the scale his or her life satisfaction as a whole rather than summing some specific domains of satisfaction with life (e.g., work, health, the sexual sphere, or marriage). Life satisfaction has been found to be one of the most important predictors of good health and successful adaptation to life [24]. The factors that may affect the increase in life satisfaction include high self-esteem, a satisfying marriage, income, and social relations, whereas unemployment, stress, anxiety, or depression may decrease life satisfaction [25,26,27,28,29]. Research indicates that people with a disability and poorer health status present lower life satisfaction than healthy controls [30,31]. Zonash et al. [32] showed that university students with acne vulgaris report lower life satisfaction than their counterparts without acne.

Krejci-Manwaring et al. [17] suggested that acne is a biopsychosocial skin condition that should be treated by considering both psychosocial and biological factors. Personality plays a vital role in predicting subjective well-being across the lifespan by promoting health-related behaviors that help or hinder adaptation to life demands [33,34]. A systematic review study demonstrated that the evaluation of both personality and health-related quality of life might aid medical research, clinical practice, and health policy evaluation [35]. Therefore, the current study examined the relationship between satisfaction with life and types of personality in young women with acne vulgaris.

Rosenman [36] described the Type A behavior pattern (TABP) as a constellation of the following characteristics: competitive striving for achievement, a strong sense of time urgency, impatience, hurried behavior, aggressiveness, ambitiousness, drive, a tendency to dominate, explosive speech stylistics, desire to control, impulsivity, restlessness, an orientation toward work responsibility, and easily provoked hostility. The opposite pattern, i.e., more relaxed, easygoing, satisfied, and unhurried, with a relative absence of drive, sense of time urgency, ambition, desire to compete, and involvement with deadlines, is named Type B, a low coronaryrisk behavior. Thomas [37] showed that Type B individuals experience less stress due to daily frustration, feel less pressured by too many tasks to complete, enjoy their life more, are more satisfied with their work, and have better general health than Type A people. Hisam et al. [38] found a rate of TABP of approximately 11% among a large sample of medical students (*n* = 500). Among Romanian university students, a high-stress level (44% of the sample), alexithymia (26%), and Type A (26%) and Type C (17%) personality patterns are much more prevalent compared to the general population, especially in medical students [39].

Type A behavior has been associated with cardiovascular disease [40], including coronary heart disease (CHD) [41,42], myocardial infarction (AMI) [43], blood pressure [44], and hypertension [45]. TABP has also been found to be related to unhealthy behavior, such as excessive alcohol drinking and cigarette smoking [46,47,48,49]. The Framingham Type A Scale (FTAS) has been found to be positively related to psychosomatic health problems, physiological overactivity to stressors, neuroticism, anxiety, and general irrational thinking, and negatively associated with self-control [50,51,52,53,54].

Type D personality refers to a distressed personality type [55]. Denollet [56] extracted two dominant traits of the Type D Scale (DS14): negative affectivity (NA) and social inhibition (SI). Individuals with high scores on the NA subscale frequently experience dysphoria, worry, and irritability and demonstrate negative feelings such as distress, dissatisfaction, anxiety, and depression. They also concentrate on the negative aspects of life. People with heightened SI typically experience discomfort in social interaction, so they tend to inhibit self-expression and exhibit reticence and a lack of social poise. There is a positive correlation between the NA subscale and neuroticism and a negative association between the SI subscale and extroversion [56]. Furthermore, a previous study found the prevalence of Type D personality to be 28% in a large sample of participants, including 21% of the general population, 28% of coronary heart disease patients, and 53% of people with a hypertension diagnosis [56]. Meanwhile, the prevalence of Type D personality in the German population has been shown to be 31% [57].

Evidence suggests that people classified as Type D personality experience physiological hyper-reactivity related to greater cortisol reactivity to stress and activation of pro-inflammatory cytokines, which may increase susceptibility to many diseases [58,59,60]. Indeed, research has shown that a distressed personality is associated with poorer quality of life [61,62,63] and various somatic symptoms and diseases [64,65,66,67,68,69], including fibromyalgia (FM) [61,70], type 2 diabetes and osteoarthritis [70], rheumatoid arthritis [70,71], chronic tinnitus [72], insomnia [73], psoriasis [74,75,76,77], and cardiovascular diseases [78,79,80], particularly myocardial infarction (MI) [81], CHD [55,58,62], and hypertension [55]. Type D personality is also related to low levels of positive affect, poor quality of life and self-esteem, high mental distress, post-traumatic stress disorder, depression, and social and general anxiety [60,63,64,65,66,68,72,76,79,80,82,83,84]. Moreover, Type D personality is positively associated with acne vulgaris [85] and negatively with life satisfaction [71,82,86,87,88].

Ferguson [89] suggested that personality traits play a central role in health processes. Personality traits are both sensitive and reactive to environmental contingencies and are associated with neurobiological processes. Therefore, they can explain an individual’s physiological, behavioral, cognitive, and cultural responses to illness. A systematic review confirmed the relationships among various aspects of personality and health-related quality of life (HRQOL) [35]. Personality can be explained by up to 45% psychosocial and 39% physical HRQOL variance. According to the model proposed by Huang et al. [35] (p. 5), personality characteristics are more strongly associated with the psychosocial aspects than the physical aspects of well-being. A pathway of personality to the psychosocial and physical aspects of health reflects the mechanisms through which personality influences HRQOL. This model hypothesizes that personality type mediates the relationship between acne severity and life satisfaction as a cognitive dimension of quality of life.

Previous research has evidenced the relationship between various diseases and TABP or Type D personality. Type D personality has been found to be a predictor of mental and physical HRQOL, depression, anxiety, and health-related distress [72]. However, research on the association between personality type and dermatologic diseases is scarce. Basińska and Woźniewicz [75] showed higher Type D personality scores among patients with psoriasis (women in particular) than the control group. Furthermore, Type D personality has been shown to be significantly associated with an impaired HRQOL and more frequent among individuals with moderate to severe psoriasis (39%) than in a control sample (24%) [76]. In a recent study, Type D personality presented in 38% of the patients with psoriasis and was associated with worse HRQOL, more sleep problems, and poor social adaptation [74]. Tekin et al. [77] found a positive correlation between Type D personality and the severity of psoriasis and a negative correlation with the quality of life (measured using the Dermatology Life Quality Index (DLQI)) in the Turkish population.

To date, there has only been one study in the field of acne vulgaris, indicating that Type D traits may be more common among patients with acne (49%) compared to those without acne (18%) [85]. Furthermore, acne patients report significantly more depressive symptoms, anxiety, social anxiety, self-reported stress, anxiety sensitivity, and disability levels than healthy controls. The levels of life satisfaction have been shown to be lower in people with acne vulgaris than in healthy university students [32], and lower in people with a Type D personality than in those without a Type D personality among both clinical and non-clinical populations [71,82,86,87,88]. However, the association between satisfaction with life and Type D personality has never been examined in people with acne vulgaris. Furthermore, to the best of our knowledge, there is also a lack of research regarding the relationship between TABP and life satisfaction among patients with acne vulgaris. Previously, TABP was discussed as a moderator of the stress response [90]; meanwhile, TABP was found to be a mediator of the relationship between stressors (life events) and mental health status (subjective stress level and the depressive symptoms) among Japanese metropolitan Tokyo residents [91].

The association between life satisfaction and personality types A and D were examined for the first time in this study among young women with acne vulgaris. TABP and Type D personality were considered in this study as categorical and continuous variables, consistent with previous research [64,73]. Recent studies [92] have shown that Type D personality effects are better modeled with continuous interaction between the two NA and SI components. Synergistic effects can be detected if two predictors (e.g., SI and NA) show a combined influence on the outcome (e.g., life satisfaction) when such effects are more than the sum of their parts. However, whether the Type D personality effect is synergistic or additive has not yet been determined. Therefore, this research explored both the synergistic and additive models of Type D personality’s impact on life satisfaction in people with acne.

Based on the vast literature to date, we formulated the following hypotheses (H):

**Hypothesis 1** **(H1).** *Satisfaction with life will be lower in women with acne vulgaris than in the female control sample without acne.*

**Hypothesis 2** **(H2).** *Women with acne vulgaris will score higher in terms of personality types A and D and will be diagnosed more frequently as Type A (compared to Type B) and Type D (compared to non-Type D) than their counterparts without acne vulgaris.*

**Hypothesis 3** **(H3).** *Type D personality and TABP are negatively associated with satisfaction with life.*

Various regression models were used to explore the relationship between acne severity and satisfaction with life, including the mediating and moderating roles of TABP and Type D personality. The moderating role of TABP in the relationship between acne severity and life satisfaction was examined in the first step (Study Model 1, Supplementary Materials: Figure S1). The moderating role of NA and SI on the association between acne severity and life satisfaction was examined using two alternative regression analyses (Study Model 2 for the additive approach and Study Model 3 for the synergistic approach, Supplementary Materials: Figures S2 and S3, respectively). Furthermore, to examine the mediating effect of Type D on young women’s life satisfaction with acne vulgaris, three alternative models were compared, including parallel mediation in Study Model 4 (Figure 2), serial mediation in Study Model 5 (Figure 3), and the synergistic approach of moderated mediation in Study Model 6 (Supplementary Materials: Figure S6). 

## 2. Materials and Methods

### 2.1. Participants

The study consisted of 300 young women studying nursing and cosmetology in the Health Science major program at one of two medical universities in Poland, ranging in age between 19 and 24 years (M = 21.28, SD = 1.39). The total sample was divided into two groups: 150 (50%) women with acne vulgaris (AV) and 150 (50%) women without acne vulgaris (WAV). The two samples were matched in age, university major, education, and gender.

### 2.2. Measures

#### 2.2.1. Acne Vulgaris

The severity of acne vulgaris was assessed using the Hellgren–Vincent Scale (HVS). In the HVS, the number of skin eruptions in people suffering from acne are classified using a five-point scale, as follows: (1) erythema, blackheads, and 1–5 pustules or papules; (2) erythema, blackheads, and 6–10 pustules or papules; (3) erythema, blackheads, and 11–20 pustules or papules; (4) erythema, blackheads, and 21–30 pustules or papules; (5) erythema, blackheads, and over 30 pustules or papules [93]. In the AV group, all participants presented with mild acne, including 96 (64%) women with first-degree acne severity and 54 (36%) females with second-degree acne severity based on the HVS. In the WAV group, none of the female students had symptoms of acne vulgaris.

#### 2.2.2. Satisfaction with Life

A global cognitive judgment of satisfaction with one’s life was assessed using a short five-item instrument, namely, the Polish version [94] of the Satisfaction with Life Scale (SWLS) [22].Respondents indicated how much they agreed or disagreed with each of the five items on a seven-point Likert-type scale ranging from 1 (Strongly Disagree) to 7 (Strongly Agree). Total scores (sum of all five items) ranged between 5 and 35, with higher scores suggesting greater life satisfaction. The scale also shows good convergent and discriminate validity with other emotional well-being scales [95]. The reliability of the SWLS is high in the Polish version [94] (Cronbach’s α = 0.81), as well as in the current study (Cronbach’s α =0.90).

#### 2.2.3. Type A Behavior Pattern

The FTAS was developed to measure a behavior pattern (e.g., emotional lability, aging worries, tension, or anger symptoms) that could predict CHD in the Framingham Heart Study [96]. The FTAS consists of 10 self-report items to assess an individual’s competitive drive, sense of time urgency, and perception of job pressure. The FTAS is divided into two parts. Five items describe such characteristics of TABP as pressed for time, hard-driving and competitive, bossy and dominating, a need to excel, and eating too quickly. Respondents answer each item using a four-point scale expressing the degree of compliance with the behavior (0 = Definitely not, 0.33 = Probably not, 0.67 = Probably, and 1 = Definitely). The second part of the questionnaire comprises five items related to work orientation and time pressure, with two answer options (0 = No and 1 = Yes). The total result is the mean of 10 items, which ranges from 0–1; average values closer to 1 indicate Type A, while values closer to 0 are interpreted as Type B personalities. Juczyński [94] suggested that TABP is confirmed if the scores are 0.5 of a standard deviation (SD) above the mean (M). Previous factor analysis showed two factors in the Polish adaptation study [94], namely, hassle (H) and rivalry (R), with five items included in each subscale. The acceptable reliability of the FTAS was reported previously in Polish samples [94], with Cronbach’s α = 0.62 and α = 0.70 [48]. In this study, α = 0.79.

#### 2.2.4. Type D Personality

The DS14 was developed to assess Type D personality [56]. The DS14 is a 14-item questionnaire that involves two subscales: negative affectivity (NA) and social inhibition (SI), each consisting of seven items. Each item is assessed on a five-point Likert-type scale (from 0 = False to 4 = True). Scores can be summarized separately in the NA and SI subscales, ranging from 0 to 28, with higher scores indicating a greater Type D personality level. Type D personality is confirmed if an individual presents with a score of ≥10 on both scales. The two-factor structure of the DS14 has been confirmed in the Polish adaptation [97], indicating an internal consistency (Cronbach’s α) of 0.86 for the NA subscale and 0.84 for the SI subscale [97].Using the standardized cut-off ≥10 for both NA and SI to identify those with a Type D personality, the prevalence has been shown to be 35% in healthy participants and 72% in patients with cardiovascular disease (CVD) [97]. In the present study, reliability coefficients (Cronbach’s α) for the NA and SI subscales were 0.72 and 0.80, respectively.

### 2.3. Procedure

The study was carried out from October 2019 to November 2019 at two universities in Poland, the Opole Medical School in Opole and the Higher Medical School in Kłodzko. The respondents completed the questionnaires (in a paper and pencil form) at the end of their lectures at university, with the lecturers’ consent. Students were made aware of the aim and content of the survey and that they could withdraw from the study at any time without providing a reason. All questionnaires were completed and returned. Written informed consent was obtained from all patients before enrollment. The Bioethics Committee of Opole Medical School in Opole approved the study protocol (no. KB/58/NOZ/2019). The study procedures were carried out in agreement with the Declaration of Helsinki.

### 2.4. Statistical Analysis

Descriptive statistics, including mean, median, standard deviation, skewness, and kurtosis, were conducted at the beginning of the statistical analysis. The differences between the AV and WAV groups were examined using Student’s t-test for satisfaction with life and personalitytypes A and D (considering them a dimensional continuous variable), and using Pearson’s χ^2^ test (with Type A and D as a categorical dichotomous variable) (i.e., the association between life satisfaction and personality types A and D). The moderating role of TABP (Study Model 1) between acne severity and life satisfaction was examined using Model 1 of PROCESS v3.3. macro for SPSS, designed by Hayes [98,99]. Two alternative models of regression analysis were used to explore the moderating effect of Type D personality on satisfaction with life: Model 2 of PROCESS v3.3 was conducted to examine the additive approach (Study Model 2), whereas Model 3 of PROCESS v3.3 was performed to test the synergistic approach (Study Model 3).Furthermore, Model 4 of PROCESS v3.3. was used to examine the parallel multiple mediating role of NA and SI on the relationship between acne severity and satisfaction with life (Study Model 4), which represents the additive approach to Type D personality. Two alternative regression analyses were performed to examine the mediating role of Type D personality in the relationship between acne severity and life satisfaction. Model 6 of PROCESS v3.3 was used to test the serial multiple effects of NA and SI (Study Model 5), whereas Model 14 of PROCESS v3.3 was used to examine the synergistic effect of NA and SI (e.g., interaction between SI and NE) in a moderated mediation analysis (Study Model 6).

The conditional effect was examined based on a bias-corrected bootstrapping procedure with 10,000 samples. A bootstrap confidence interval (95% CI) not including “0” signals a significant effect. Moreover, as Preacher et al. [100] suggested, the independent variable was mean-centered before analysis to provide a clearer and easier explanation of the interaction effect between the predictor and moderator variables on the dependent variable. All analyses were performed using Statistical Package for the Social Sciences (IBM SPSS Statistics, ver. 25, 2019, Predictive Solutions Sp. z o.o., Kraków, Poland).

## 3. Results

### 3.1. Differences in Satisfaction with Life andPersonality Types A and D Between the WAV and AV Groups

The descriptive statistics, including range, M, and SD (median, skewness, and kurtosis), are shown in Table 1 for the dimensional continuous variables of the SWLS, the FTAS, and the DS14. The kurtosis and skewness values ranged between ±1.0, which may be considered to indicate excellent psychometric characteristics [101]. Thus, parametric tests were conducted in further statistical analyses. Student’s *t*-test was used to compare the WAV and AV samples in terms of life satisfaction, TABP, and Type D personality scores (Table 2). The AV group reported significantly lower levels of satisfaction with life than the WAV sample, confirming hypothesis H1. Moreover, the AV individuals showed higher TABP and Type D personality than the WAV female students. This was in line with hypothesis H2 regarding the approach to personality type as a continuous variable.

A comparison of the prevalence of satisfaction with life, TABP, and Type D personality in the WAV and AV samples is shown in Table 3. As the contingency table indicates, in the group of women with acne, there was a statistically significant number of individuals not satisfied with life and who fulfilled the criteria for personality types A and D than in the group of females without acne. Therefore, the H2 hypothesis can also be confirmed when the personality type is considered a dichotomous variable.

### 3.2. Association Between Satisfaction with Life and Personality Types A and D in Women with Acne 

Hypothesis H3 was examined using a preliminary correlation analysis and further regression to explore the moderating and mediating roles of personality types on satisfaction with life (SWL) in young women with acne vulgaris. Pearson’s correlation of life satisfaction with personality types A and D are shown in Table 4. A weaker negative correlation was found in the WAV group between life satisfaction and the NA and SI subscales of the DS14 than in the AV sample. TABP was found to be unrelated to satisfaction with life as a dimensional variable. However, TABP differed significantly in the WAV and AV groups, as was demonstrated previously. Thus, the moderating role of Type A personality in the relationship between acne severity and satisfaction with life was further examined in the following analysis. For this purpose, TABP was used as a categorical, dichotomous variable (coded 0 = NoTABP and 1 = TABP), whereas acne severity was assessed as a multi-categorical variable from the HVS (coded 0 = WAV, 1 = first-degree acne severity, and 2 = second-degree acne severity). 

**Figure 1:**
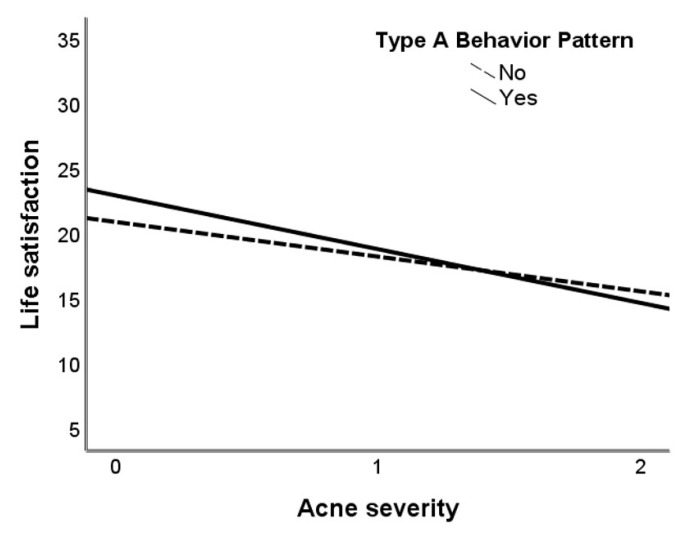
An interaction effect between acne severity and TABP on satisfaction with life.

The regression analysis results showed an interaction effect between second-degree acne severity and TABP on life satisfaction, with a medium effect size (Table 5, Figure 1, Supplementary Materials: Figure S1). Although the *p*-value was 0.055, the bootstrap results for the regression model parameters were: Boot *b* = −4.12, M = −4.11, Boot *SE* = 2.03, and Boot 95% CI(−8.12, −0.05), which may be interpreted as a significant interaction effect between TABP and acne severity on satisfaction with life. Study Model 1 explained no more than 14% of satisfaction with life variance. Similar moderation results were found when TABP was considered a continuous variable: *R*^2^ = 0.15, *F*(5, 294) = 10.165, *p* < 0.001 (see Supplementary Materials for more details). Alternative mediation analysis was not performed because TABP did not correlate with the dependent variable (i.e., life satisfaction) and therefore could not be considered a predictor. 

The moderating role of NA and SI as two sub-dimensions of Type D personality was examined in both approaches: additive (Study Model 2, Supplementary Materials: Figure S2) and synergistic (Study Model 3, Supplementary Materials: Figure S3). Moderation analysis revealed an interaction effect between SI and first-degree acne severity. However, no interaction effect between NA and AV was found in Study Model 2: *R*^2^ = 0.22, *F*(8, 291) = 10.18, *p* < 0.001 (see Supplementary Materials for more details). Moreover, taking into account the synergistic approach, the interaction effect was found solely for SI and first-degree acne severity, but not between SI and NA or between SI and NA: *R*^2^ = 0.23, *F*(11, 288) = 7.62, *p* < 0.001. These results indicate that the moderation model of regression cannot sufficiently explain the variance in the association between life satisfaction and Type D personality in women with acne. Moreover, a synergistic effect of NA and SI on life satisfaction was not found in this research.

**Figure 2:**
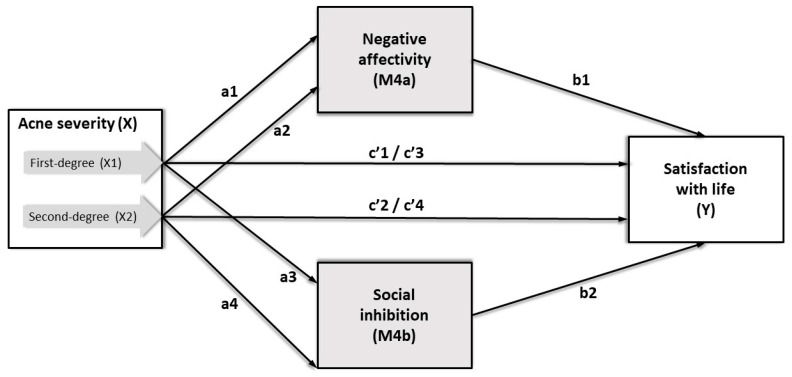
Parallel multiple mediation model for predicting satisfaction with life (Study Model 4).

Two parallel multiple mediation analyses were conducted to examine the mediating role of both subscales of the DS14 (i.e., NA and SI) on the relationship between acne severity and satisfaction with life (Figure 2). The results of the Study Model 4a mediation analysis (Table 6, Figure S4, and Supplementary Materials) indicated that NA (M1) completely mediated the relationship between first-(X1) and second-degree (X2) acne severity and life satisfaction (Y). Female students with greater acne severity and higher scores in the NA subscale of the DS14 were less satisfied with life. The relative indirect effect of first-degree acne severity on satisfaction with life for negative affectivity as a mediator equaled Boot *b* = −1.59, Boot *SE* = 0.43, and Boot 95% CI(−2.43, −0.73). The relative indirect effect of second-degree acne severity on satisfaction with life for negative affectivity as a mediator equaled Boot *b* = −1.89, Boot *SE* = 0.55, and Boot 95% CI(−3.00, −0.83). The total variance of life satisfaction accounted for by Study Model 4a was 18%.

Furthermore, the results of the Study Model 4b mediation analysis (Figure 2, Table 6, and Supplementary Materials) indicated that SI (M4b) completely mediated the relationship between first- (X1) and second-degree (X2) acne severity and satisfaction with life (Y). Participants with greater acne severity and higher scores in social inhibition were less satisfied with life. The relative indirect effect of first-degree acne severity on satisfaction with life via social inhibition equaled Boot *b* = −1.62, Boot *SE* = 0.43, and Boot 95% CI(−2.50, −0.83). The relative indirect effect of second-degree acne severity on satisfaction with life via social inhibition equaled Boot *b* = −2.11, Boot *SE* = 0.59, and Boot 95% CI(−3.39, −1.06). The total variance of life satisfaction accounted for by Model 2 was also 18%.

**Figure 3:**
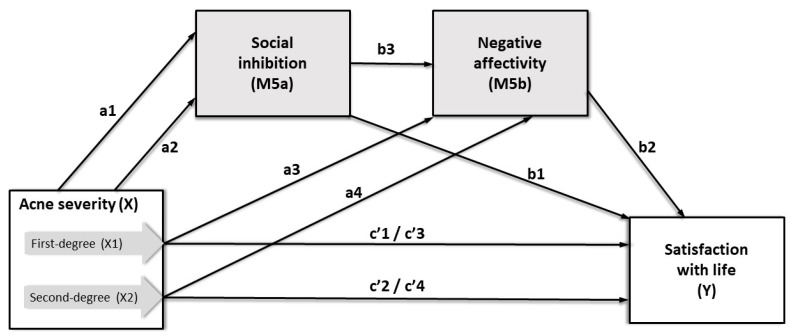
Serial multiple mediation model for predicting satisfaction with life (Study Model 5).

The additive effect of the SI and NA subscales of the DS14 on life satisfaction was examined using Study Model 5 (Figure 3, Table 7, Figure S5, and Supplementary Materials), whereas the synergistic approach, with the combined (interacted) influence of SI and NA, was tested using Study Model 6 (Supplementary Materials: Figure S6). 

According to previous research [102], social inhibition modulates the effect of negative emotions on cardiac prognosis. Therefore, SI preceded NA in Study Model 5. The results of the Study Model 5 mediation analysis showed that both SI (M5a) and NA (M5b) completely mediated the relationship between first-(X1) and second-degree (X2) acne severity and satisfaction with life (Y). The relative indirect effect of first-degree acne severity on life satisfaction via SI and NA equaled Boot *b* = −0.43, Boot *SE* = 0.0,21, and Boot 95% CI(−0.82, −0.36). The relative indirect effect of second-degree acne severity on life satisfaction via SI and NA equaled Boot *b* = −0.57, Boot *SE* = 0.28, and Boot 95% CI(−1.13, −0.05). The total variance of life satisfaction accounted for by Study Model 5 increased to 20% compared to Study Model 4. Study Model 6 also explained 20% of life satisfaction variance, but moderated mediation was not confirmed, since the interaction coefficient was Boot *b* = 0.02, Boot *SE* = 0.01, and Boot 95% CI(−0.02, 0.03). Study Model 5 of the synergistic approach explained the higher percentage of life satisfaction variance and best fit the data compared to Study Models 2–4 and 6. More details are available in Supplementary Materials.

## 4. Discussion

This study aimed to explore, for the first time, the association and possible mechanism of the relationship between acne severity and satisfaction with life as a dimension of quality of life, as well as personality types A and D. Consistent with hypothesis H1 and a previous study [32], young women with acne vulgaris reported a lower level of satisfaction with life than their counterparts without acne. Moreover, this research suggests that the prevalence of women satisfied with their life is much higher in the WAV group than the AV group. Indeed, poorer health and disability usually decrease life satisfaction [30,31], and people suffering from severe symptoms of acne typically show a low quality of life [13,14,15,16,17,18,19,20,33]. Wan [103] suggested that stress may aggravate acne by inducing inflammatory cytokines and cortisol production, causing higher inflammation levels. Previous research showed that individuals with acne experience high stress, anxiety, and depression [9,10,11,12], which may also decrease subjective well-being and satisfaction with life [25,26,27,28,29]. In turn, long-term stress reduces life satisfaction and contributes to the emergence of emotional and mental health problems [29]. Subjective well-being and mental and somatic health are bi-directionally related to one another.

Hypothesis H2 that female university students with AV will score higher in terms of personality types A and D than the WAV group was confirmed. Furthermore, women meeting the TABP and Type D personality criteria were significantly more likely to be found in the AV sample rather than the WAV group. It is important to note that TABP was examined for the first time among people with acne vulgaris in this study. Per the association between TABP and unhealthy behavior, various somatic diseases, and mental problems [36,40,41,42,43,44,45,46,47,48,49,50,51,52,53,54], individuals with acne vulgaris demonstrated significantly higher TABP than those without acne in this study. An individual with a Type A personality is engaged in an excessive struggle to obtain countless things from his environment in the shortest period of time. People with TABP usually tend more to negative feedback, believing their performance is never good enough and experiencing a more significant number of stressful encounters and higher occupational stress. TABP has been identified as a predisposing factor to both increased stress and a maladaptive coping response to stress [47,104]. TABP has been found in11% of medical students [38] compared to 26% in university students [39]. The prevalence of TABP in the present sample was approximately 12% among female students without acne and 24% in the AV sample. Thus, we can assume that the prevalence of acne in the present study is consistent with previous research.

Consistent with hypothesis H2, the present findings indicate that women with acne have higher Type D personality scores than their counterparts without acne. This result is also consistent with a previous study regarding acne [85] and the extensive scientific literature on the relationship between Type D personality and various somatic diseases and mental problems or disorders [62]. Moreover, the findings suggest that the relationship between Type D personality and health outcomes may be generalized across different chronic illnesses [70]. A recent study found multiple possible biological and behavioral pathways between Type D personality and increased morbidity and mortality [105]. People with a Type D personality demonstrate weaker glycemic control, systemic inflammation, and poorer autonomic nervous system modulation. They also report less social support and greater sleep difficulties than those without a Type D personality. People with a Type D personality are more likely to report cardiac–sympathetic, metabolic, vasovagal, muscular, and headache symptoms, poorer health, increased minor illnesses, work absences, and medical information seeking than people without a Type D personality [64].

Furthermore, stressful events and anxiety are mediators of the relationship between Type D personality and physical symptoms. Previous review studies have shown that Type D personality is associated with a low physical and mental health status and poor self-management of the disease [67,68]. The review studies also found a relationship with an increased number or severity of reported physical health complaints, including chronic pain, asthma, vertigo, influenza-like illness, and general health status. The negative effect of Type D personality on work-related problems has also been shown regarding higher absence/leave, higher levels of vital exhaustion and burnout, and more work-related stress. Type D personality has also been linked positively to passive coping and negatively to social support, health-related behaviors, adherence to treatment, and effort to perform diagnostic testing. In general, Type D personality increases the risk of mental distress [106].

In the present research, the prevalence of Type D personality was approximately 16% among women without acne and 41% in the AV sample, indicating an average of 28% for university students. This result is largely in line with previous research [56,57,85]. Sereflican et al. [85] found Type D personality (as a dichotomous classification) among 49% of Turkish patients with acne and in 18% of healthy controls without acne. However, in another study, Type D personality was confirmed in 44% of Iranian university students [86]. The different prevalence rates of Type D personality between particular studies may be related to cross-cultural differences [79]. For our Polish sample, an average NA score of 10 (M = 9.7, SD = 6.9, Cronbach’s α = 0.89) and a mean SI result of 9 (M = 9.0, SD = 5.8, Cronbach’s α = 0.80) were obtained, whereas the prevalence of Type D personality was previously indicated to be 35% in Eastern European countries [79]. Comparatively, in the present sample, we found higher average scores among female university students in both subscales of the DS14: NA (M = 14.8, SD = 6.2) and SI (M = 11.2, SD = 5.8). The differences in Type D personality may be related to the specific population of university students, which was found to be a higher risk of stress, anxiety, and depression than the general population [107]. 

Contrary to hypothesis H3, TABP, as a dimensional continuous variable, was found not to be related to satisfaction with life in this study. However, using TABP as both a categorical dichotomous variable and as a continuous variable, we demonstrated that Type A personality may play a moderating role between acne severity and satisfaction with life. Therefore, the moderating role of TABP confirms hypothesis H3. TABP was previously considered a moderator of the stress response [90]. In the current sample of young female university students without acne, higher levels of TABP were related to higher life satisfaction. Conversely, among women with second-degree acne severity, those with higher TABP were less satisfied with life. It is likely that acne severity is an essential factor that determines the interaction with Type A personality and affects life satisfaction. Furthermore, a longitudinal study showed that global Type A behavior increased from adolescence to adulthood. Hintsa et al. [108] suggested that both life span and societal changes should be considered concurrently to fully understand the health consequences of TABP. Future research on the relationship between TABP and satisfaction with life should be conducted in people with acne of various ages from adolescence to late adulthood. More research is also necessary particularly among groups with higher acne severity levels than is presented in this study.

Consistent with hypothesis H3, the present findings indicate a negative correlation of life satisfaction with both subscales of the DS14, namely, negative affectivity and social inhibition. This result confirms previous studies that found a negative association between satisfaction with life and Type D personality [71,82,86,87,88]. Type D personality has been shown to be correlated with elevated stress, anxiety, depression, and low quality of life [80,84], as well as a predictor of self-reported physical symptoms, stress, and anxiety [69]. Moreover, as a Type D personality component, negative affectivity has been indicated as a predictor of depression, anxiety, mood (both positive and negative), social phobia, and loneliness. Meanwhile, in another study, social inhibition was shown to be a predictor of general anxiety, social phobia, positive mood (negatively), emotional and behavioral inhibition, and loneliness among patients with coronary artery disease [109].

This study showed that the association between Type D personality and satisfaction with life was stronger among the AV group compared to the WAV group. As indicated earlier, higher stress and mental disturbance have been found in people with acne than in controls without acne [9,10,11,12], which may affect life satisfaction to a greater extent [25,26,27,28,29] and seems to explain the present findings. In previous research, dispositional social sensitivity understood as a personality trait was associated with the adverse social impact of acne in a large sample of people between the ages of 16 and 62 [17]. Other research has indicated that people with acne have difficulties in emotion regulation [14]. Furthermore, greater acne severity is significantly associated with lower quality of life and social functioning [2,3,4,5,6,7,8,9,10,11,12].

Finally, the moderating role of NA and SI was examined compared to both the additive and synergistic approaches of Type D personality. Moderation analysis showed no interaction between NA and acne vulgaris severity (AVS) and a weak interaction effect of SI and first-degree acne severity on life satisfaction in both the additive and synergistic approaches. As emphasized in previous research [92], synergy can be considered if an interaction effect between the two predictors can improve the regression model. Because an interaction between continuous NE and SI (as both subcomponents of the DS14) was not found in either Study Model 3 of the moderation analysis or in Study Model 6 in the moderated mediation analysis, the synergistic approach cannot be supported in the present research. Previous research demonstrated that Type D personality is better represented as a dimensional construct than a categorical variable [33]. Furthermore, Horwood et al. [70] showed that superior prediction of health outcomes might be achieved using NA and SI as independent subscales. Their study also showed that Type D personality (NA and SI interaction) is related to significantly higher depression and mood than NA or SI separately [109]. In contrast, another study [110] indicated that an additive approach to Type D personality may be more valuable than a synergistic approach. The NA subscale in the study was a significant predictor of poorer quality of life (QoL) among patients with post-myocardial infarction, but the continuous interaction approach (combined NA and SI) did not support a relationship. Moreover, Lodder [92] showed in his re-analyses that the continuous interaction approach failed to reach significance. Therefore, both the present and some previous studies do not support the synergistic effect of Type D personality.

Regarding the additive approach to Type D personality, the parallel multiple mediation analysis of Study Model 4 showed that both the negative affectivity and the social inhibition subscales of the DS14 mediate the relationship between acne severity and life satisfaction. Female students with greater acne severity and higher negative affectivity and social inhibition were less satisfied with life. However, it is essential to note that the total variance of life satisfaction accounted for by Study Model 4a and Study Model 4b was only 18%. Thus, the other variables not considered in this study could be more significant than acne severity and life satisfaction. 

In contrast, the serial multiple mediation analysis of Study Model 5 seemed more appropriate for AVS. Study Model 5 explained 20% of the variance, and both SI and NA were found to be significant mediators of the association between acne severity and life satisfaction. Both the NA and SI subcomponents of the DS14 seemed to share a large amount of the variance. The mediation model increased by only 2% in the life satisfaction explanation in Study Model 5 if NA and SI were considered simultaneously. In comparison, when NA and SI were considered separately in the mediation model, each variable explained 18%of the life satisfaction variance in Study Model 4. It is important to note that an interaction between SI and NA was not confirmed in the moderated mediation Study Model 6.However, further research is needed to support the findings.

### Study Limitations

There are some limitations of this study. First of all, the cross-sectional design restrained us from drawing any conclusions on causality. Additionally, the study results cannot be generalized to the male population and individuals in different developmental periods, such as adolescence or those in medium and late adulthood. Moreover, some biased answers may have resulted from the self-report measures for assessing life satisfaction and personality types A and D. Future studies should consider a longitudinal experimental design with a much larger sample of various ages, and with an equal number of male and female representatives of the general population.

## 5. Conclusions

The relationship between life satisfaction and personality types A and D was examined in this study for the first time in female university students with acne vulgaris. This study suggested that personality types A and D are presented more frequently in young adult women with acne vulgaris than in those without acne vulgaris. Women with acne vulgaris reported higher levels of personality types A and D and lower life satisfaction. Type A personality was shown to play a moderating role, whereas both the NA and SI subcomponents of the DS14 showed a mediating effect on the relationship between acne severity and life satisfaction. Young women with TABP were more satisfied with their life when they did not exhibit symptoms of acne. In contrast, young female adults with symptoms of Type A personality and second-degree AVS were concurrently less satisfied with life. Thus, the tendency to experience high stress with regard to acne symptoms may lead to a decrease in life satisfaction. Rout and Rout [90] recommended several coping strategies that may help manage stress and TABP effectively to reduce health-related risky behavior, including quietly listening to others, thinking before saying something, changing obsessional time-directed behavior, carrying out exercises to assess Type A behavior, slowing down, widening outside activities, avoiding making an unnecessary appointment and protecting a time, practicing stress-free breathing, and appreciating others.

Furthermore, this study highlighted that high levels of negative emotionality and social inhibition as two subcomponents of the DS14 lead to decreased satisfaction with life among women with acne symptoms. The present study results did not confirm the synergistic effect of Type D personality on satisfaction with life. Both of the NA and SI subscales, included simultaneously in the serial multiple mediation model, explained 20% of the life satisfaction variance. Regression analysis showed the significant mediating effect of both of the NA and SI subscales on the relationship between acne severity and life satisfaction. Therefore, we can conclude that Type D personality explains the mechanism of association between acne disease and subjective well-being. Unfortunately, a recent review did not find any publications regarding psychological interventions for Type D personality [62]. Kupper and Denollet [62] suggested that stepwise psychotherapy to improve depressive symptoms or intervention programs for health-related psychological problems may improve mood, quality of life, and physical functioning of people with a Type D personality who suffer from the somatic disease. Furthermore, Smith et al. [111] showed the efficacy of positive emotional writing as a helpful intervention for alleviating the adverse psychological effects of Type D personality (like stress and anxiety) in the general population.

## Figures and Tables

**Table 1:** Descriptive statistics.

Variable	Range	M	SD	Median	Skewness	Kurtosis
Satisfaction with life	5–35	19.36	6.86	18.00	0.15	−0.53
Type A behavior pattern	0–1	0.67	0.23	0.73	−0.86	0.32
TypeD personality						
Negative Affectivity	0–28	14.77	6.23	16.00	−0.55	−0.32
Social inhibition	0–28	11.15	5.84	11.00	−0.02	−0.38

**Table 2:** Differences between the without acne vulgaris (WAV) and acne vulgaris (AV) samples in terms of life satisfaction, Type A behavior pattern (TABP), and Type D personality.

Variable	WAV		AV		*t* (298)	*p*	*d*
	M	SD	M	SD			
Satisfaction with life	21.57	7.24	17.14	5.66	5.91	0.000	0.68
Type A personality	0.57	0.26	0.77	0.14	−8.27	0.000	0.96
Type D personality							
Negative Affectivity	11.88	6.46	17.66	4.39	−9.07	0.000	1.05
Social inhibition	8.51	5.64	13.79	4.75	−8.77	0.000	1.01

**Table 3:** The prevalence of satisfaction with life, TABP, and Type D personality in samples of young women with (AV) and without (WAV) acne vulgaris.

Variable	WAV		AV		χ^2^(1)	*p*	ϕ
	n	%	n	%			
Life satisfaction					43.88	0.000	−0.38
No (SWLS < 20)	55	18.33	112	37.33			
Yes (SWLS ≥ 20)	95	31.67	38	12.67			
Type A behavior pattern					19.89	0.000	0.26
No (FTAS < 0.79)	115	38.33	78	26.00			
Yes (FTAS ≥ 80)	35	11.67	72	24.00			
Type D personality					76.22	0.000	0.50
No (NA ≤ 10; SI ≤ 10)	103	34.33	28	9.33			
Yes (NA ≥ 10; SI ≥ 10)	47	15.67	122	40.67			

Abbreviations: SWLS, Satisfaction with Life Scale; FTAS, Framingham Type A Scale; NA, negative affectivity; SI, social inhibition; WAV, without acne vulgaris; AV, acne vulgaris.

**Table 4:** Correlation of life satisfaction with personality types A and D.

Variable	Samples		
	Total	WAV	AV
Type A behavior pattern	−0.06	0.14	−0.01
Type D personality			
Negative affectivity	−0.37 ***	−0.23 **	−0.31 ***
Social inhibition	−0.38 ***	−0.19 *	−0.41 ***

Abbreviations: WAV, sample without acne vulgaris; AV sample with acne vulgaris.* *p*< 0.05; ** *p*< 0.01; *** *p*< 0.001.

**Table 5:** Results of the moderation analysis for satisfaction with life as a dependent variable, acne severity as an independent variable, and dichotomous TABP as a moderator.

Variable					Bootstrap 95% *CI*			
	*b*	*SE*	*t*	*p*	*M*	*SE*	*LL*	*UL*
Constant	21.26	0.60	35.59	0.000	21.26	0.68	19.90	22.59
Acne severity (AS)								
First-degree on the HVS (AS 1)	−4.50	1.08	−4.17	0.000	−4.49	0.94	−6.31	−2.63
Second degree on the HVS (AS 2)	−4.26	1.37	−3.11	0.002	−4.26	1.32	−6.79	−1.60
Type A behavior pattern (TABP)	1.34	1.24	1.08	0.280	1.34	1.43	−1.58	4.04
Interaction term AS × TABP								
Int 1 AS 1 × TABP	1.30	1.80	0.72	0.472	1.29	1.82	−2.18	4.95
Int 2 AS 2 × TABP	−4.12	2.14	−1.93	0.055	−4.11	2.03	−8.12	−0.05

Abbreviations: *SE*, standard error; *LL*, lower level; *UL*, upper level; *CI*, confidence interval. Number of bootstrap samples for the percentile bootstrap confidence intervals was 10,000. *R*^2^ = 0.14, *F*(5, 294) = 9.70, *p* < 0.001, *f*^2^ = 0.16.

**Table 6:** Path coefficients of acne severity (X1, X2), negative affectivity (M4a), and social inhibition (M4b) on satisfaction with life (Y) in a parallel multiple mediation model.

Antecedent Study Model 4a (M4a)		Consequent						
	Estimate	M4a (Negative Affectivity)			Estimate	Y (Satisfaction with Life)		
		Coefficient	*SE*	*p*		Coefficient	*SE*	*p*
X1 (first-degree AV)	a1	5.41	0.72	0.000	c’1	−1.98	0.89	0.027
X2 (second-degree AV)	a2	6.44	0.88	0.000	c’2	−4.07	1.08	0.000
M4a (negative affectivity)		-	-	-	b1	−0.29	0.07	0.000
Constant	i_M1_	11.88	0.45	0.000	i_Y_	25.06	0.93	0.000
		*R*^2^ = 0.22				*R*^2^ = 0.18		
		*F*(2, 297) = 41.71 *p* < 0.001				*F*(3, 296) = 20.86 *p* < 0.001		
**Study Model 4b (M4b)**		**M4b (Social Inhibition)**				**Y (Satisfaction with Life)**		
		**Coefficient**	***SE***	***p***		**Coefficient**	***SE***	***p***
X1 (first-degree AV)	a3	4.74	0.68	0.000	c’3	−1.97	0.88	0.025
X2 (second-degree AV)	a4	6.23	0.83	0.000	c’4	−3.86	1.08	0.000
M4b (social inhibition)		-	-	-	b2	−0.34	0.07	0.000
Constant	i_M2_	8.51	0.42	0.000	i_Y_	24.45	0.78	0.000
		*R*^2^ = 0.21				*R*^2^ = 0.18		
		*F*(2, 297) = 40.13 *p* < 0.001				*F*(3, 296) = 22.26 *p* < 0.001		

*Note.* The path a represents the impact of the independent variable (X) on the mediator variable (M). Path b represents the impact of M on the dependent variable (Y). Path c’ represents the direct effect of X on Y and is calculated controlling for the indirect, mediated effect.

**Table 7:** Path coefficients of acne severity (X1, X2), social inhibition (M5a), and negative affectivity (M5b) on satisfaction with life (Y) in a serial multiple mediation model.

Antecedent Study Model 5a (M5a)		Consequent						
	Estimate	M5a (Social Inhibition)			Estimate	Y (Satisfaction with Life)		
		Coefficient	*SE*	*p*		Coefficient	*SE*	*p*
X1 (first-degree AV)	a1	4.74	0.68	0.000	c’1	−3.57	0.84	0.000
X2 (second-degree AV)	a2	6.23	0.83	0.000	c’2	−5.96	1.03	0.000
M5a (social inhibition)	-	-	-	-	b1	−0.24	0.08	0.002
Constant	i_M1_	8.51	0.42	0.000	i_Y_	21.57	0.53	0.000
		*R*^2^ = 0.21				*R*^2^ = 0.12		
		*F*(2, 297) = 40.13 *p* < 0.001				*F*(2, 297) = 20.04 *p* < 0.001		
**Study Model 5b (M5b)**		**M5b (Negative Affectivity)**				**Y (Satisfaction with Life)**		
		**Coefficient**	***SE***	***p***		**Coefficient**	***SE***	***p***
X1 (first-degree AV)	a3	3.11	0.69	0.000	c’3	−1.38	0.90	0.120
X2 (second-degree AV)	a4	3.40	0.85	0.000	c’4	−3.22	1.10	0.004
M5a (social inhibition)	b3	0.49	0.06	0.000	-	-	-	-
M5b (negative affectivity)		-	-	-	b2	−0.19	0.07	0.000
Constant	i_M2_	7.74	0.62	0.000	i_Y_	25.90	0.96	0.011
		*R*^2^ = 0.38				*R*^2^ = 0.20		
		*F*(3, 296) = 61.22 *p* < 0.001				*F*(4, 295) = 18.66 *p* < 0.001		

*Note*. The path a represents the impact of the independent variable (X) on the mediator variable (M). Path b represents the impact of M on the dependent variable (Y). Path c’ represents the direct effect of X on Y and is calculated controlling for the indirect, mediated effect.

## Supplementary Materials

The following are available online at https://www.mdpi.com/1660-4601/17/22/8524/s1, Figure S1: Study Model 1, Figure S2: Study Model 2; Figure S3: Study Model 3; Figure S4: Study Model 4; Figure S5: Study Model 5, Figure S6: Study Model 6.
