# Supplementary material for: Association between Satisfaction with Life and Personality Types A and D in Young Women with Acne Vulgaris
Source: Int J Environ Res Public Health. 2020 Nov 17;17(22):8524. doi: 10.3390/ijerph17228524 (PMC7698541; doi:10.3390/ijerph17228524)
Supplement: Supplementary file 1 [file ijerph-17-08524-s001.pdf]

SUPPLEMENTARY MATERIALS

Study Models of the relationship between Type A and D personality and life satisfaction in young women with acne vulgaris:

Results of moderation and mediation analyses using PROCESS 3.3. software for SPSS

In: Chilicka, K.; Rogowska, A.M.; Szyguła, R.; Adamczyk, E. (2020). Association between satisfaction with life and personality type A and D in young women with acne vulgaris, *Int J Env Res Ment Health.*

STUDY MODEL 1

Moderation effect of Type A personality on the relationship between acne severity and satisfaction with life

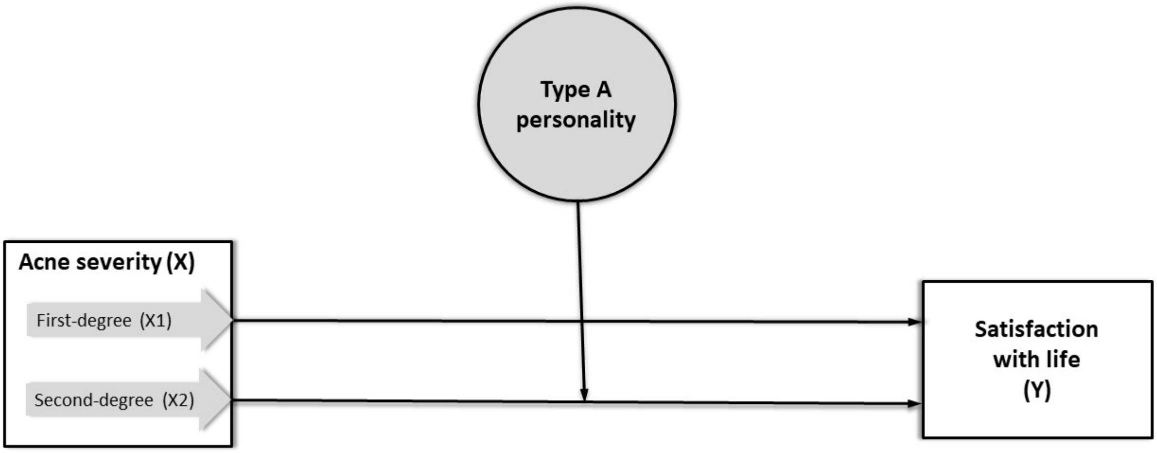

Figure S1. Study Model 1

STUDY MODEL 1a

Type A personality as a categorical dichotomous variable

**************** PROCESS Procedure for SPSS Version 3.4.1 ****************
Written by Andrew F. Hayes, Ph.D. www.afhayes.com
Documentation available in Hayes (2018). www.guilford.com/p/hayes3
****

Model : 1
Y : SWLS
X : HVS
W : TypeA
Sample Size: 300
Coding of categorical X variable for analysis:

HVS	X1	X2
.000	.000	.000
1.000	1.000	.000

2.000 .000 1.000

OUTCOME VARIABLE: SWLS

Model Summary

R	R-sq	MSE	F	df1	df2	p
.376	.142	41.038	9.696	5.000	294.000	.000

Model

	coeff	se	t	p	LLCI	ULCI
constant	21.738	.545	39.899	.000	20.666	22.811
X1	-4.034	.864	-4.671	.000	-5.734	-2.334
X2	-5.729	1.058	-5.415	.000	-7.811	-3.647
TypeA	1.339	1.237	1.083	.280	-1.095	3.773
Int_1	1.296	1.802	.719	.472	-2.250	4.842
Int_2	-4.117	2.138	-1.926	.055	-8.324	.090

Product terms key:

Int_1	:	X1	x	TypeA
Int_2	:	X2	x	TypeA

Covariance matrix of regression parameter estimates:

	constant	X1	X2	TypeA	Int_1	Int_2
constant	.297	-.297	-.297	.189	-.189	-.189
X1	-.297	.746	.297	-.189	-.004	.189
X2	-.297	.297	1.119	-.189	.189	-.247
TypeA	.189	-.189	-.189	1.529	-1.529	-1.529
Int_1	-.189	-.004	.189	-1.529	3.246	1.529
Int_2	-.189	.189	-.247	-1.529	1.529	4.569

Test(s) of highest order unconditional interaction(s):

	R2-chng	F	df1	df2	p
X*W	.018	3.162	2.000	294.000	.044

Focal predict: HVS (X)

Mod var: TypeA (W)

Conditional effects of the focal predictor at values of the moderator(s):

Moderator value(s): TypeA -.357

	Effect	se	t	p	LLCI	ULCI
X1	-4.496	1.078	-4.172	.000	-6.617	-2.375
X2	-4.261	1.370	-3.110	.002	-6.957	-1.565

Test of equality of conditional means

F	df1	df2	p
11.048	2.000	294.000	.000

Estimated conditional means being compared:

HVS	SWLS
.000	21.261
1.000	16.765
2.000	17.000

Moderator value(s): TypeA .643

	Effect	se	t	p	LLCI	ULCI
X1	-3.200	1.444	-2.216	.027	-6.041	-.359

X2	-8.378	1.641	-5.106	.000	-11.607	-5.148

Test of equality of conditional means

F	df1	df2	p
13.098	2.000	294.000	.000

Estimated conditional means being compared:

HVS	SWLS
.000	22.600
1.000	19.400
2.000	14.222

****

*********** BOOTSTRAP RESULTS FOR REGRESSION MODEL PARAMETERS ************

OUTCOME VARIABLE: SWLS

	Coeff	BootMean	BootSE	BootLLCI	BootULCI
constant	21.738	21.731	.621	20.467	22.921
X1	-4.034	-4.027	.828	-5.604	-2.371
X2	-5.729	-5.723	1.002	-7.639	-3.748
TypeA	1.339	1.351	1.406	-1.482	4.005
Int_1	1.296	1.267	1.806	-2.304	4.853
Int_2	-4.117	-4.131	2.020	-8.123	-.104

********************** ANALYSIS NOTES AND ERRORS ************************

Level of confidence for all confidence intervals in output: 95.0000

Number of bootstrap samples for percentile bootstrap confidence intervals: 10000

NOTE: The contrast option is not available with a multicategorical X.

NOTE: The following variables were mean centered prior to analysis: TypeA

STUDY MODEL 1b

Type A personality as a continuous variable

**************** PROCESS Procedure for SPSS Version 3.4.1 *****************

Written by Andrew F. Hayes, Ph.D. www.afhayes.com

Documentation available in Hayes (2018). www.guilford.com/p/hayes3

****

Model : 1
Y : SWLS
X : HVS
W : FTAS
Sample Size: 300

Coding of categorical X variable for analysis:

HVS	X1	X2
.000	.000	.000
1.000	1.000	.000
2.000	.000	1.000

****

OUTCOME VARIABLE: SWLS

Model Summary

R	R-sq	MSE	F	df1	df2	p
.392	.153	40.474	10.652	5.000	294.000	.000

Model

	coeff	se	t	p	LLCI	ULCI
constant	21.966	.557	39.452	.000	20.870	23.062
X1	-4.657	.951	-4.896	.000	-6.529	-2.785
X2	-4.674	1.259	-3.712	.000	-7.152	-2.195
FTAS	3.906	1.994	1.959	.051	-.019	7.832
Int_1	3.465	4.868	.712	.477	-6.116	13.046
Int_2	-18.843	6.746	-2.793	.006	-32.119	-5.567

Product terms key:

Int_1	:	X1	x	FTAS
Int_2	:	X2	x	FTAS

Covariance matrix of regression parameter estimates:

	constant	X1	X2	FTAS	Int_1	Int_2
constant	.310	-.310	-.310	.400	-.400	-.400
X1	-.310	.905	.310	-.400	-1.448	.400
X2	-.310	.310	1.586	-.400	.400	-4.274
FTAS	.400	-.400	-.400	3.978	-3.978	-3.978
Int_1	-.400	-1.448	.400	-3.978	23.700	3.978
Int_2	-.400	.400	-4.274	-3.978	3.978	45.502

Test(s) of highest order unconditional interaction(s):

	R2-chng	F	df1	df2	p
X*W	.026	4.461	2.000	294.000	.012

Focal predict: HVS (X)

Mod var: FTAS (W)

Conditional effects of the focal predictor at values of the moderator(s):

Moderator value(s): FTAS -.233

	Effect	se	t	p	LLCI	ULCI
X1	-5.464	1.693	-3.227	.001	-8.797	-2.132
X2	-.282	2.460	-.114	.909	-5.122	4.559

Test of equality of conditional means

F	df1	df2	p
5.217	2.000	294.000	.006

Estimated conditional means being compared:

HVS	SWLS
.000	21.055
1.000	15.591
2.000	20.774

Moderator value(s): FTAS .000

	Effect	se	t	p	LLCI	ULCI
X1	-4.657	.951	-4.896	.000	-6.529	-2.785

	Effect	se	t	p	LLCI	ULCI
X2	-4.674	1.259	-3.712	.000	-7.152	-2.195

Test of equality of conditional means

F	df1	df2	p
15.185	2.000	294.000	.000

Estimated conditional means being compared:

HVS	SWLS
.000	21.966
1.000	17.309
2.000	17.292

Moderator value(s): FTAS .233

	Effect	se	t	p	LLCI	ULCI
X1	-3.849	1.232	-3.125	.002	-6.273	-1.425
X2	-9.066	1.437	-6.308	.000	-11.894	-6.237

Test of equality of conditional means

F	df1	df2	p
20.103	2.000	294.000	.000

Estimated conditional means being compared:

HVS	SWLS
.000	22.876
1.000	19.027
2.000	13.811

****

*********** BOOTSTRAP RESULTS FOR REGRESSION MODEL PARAMETERS ************

OUTCOME VARIABLE: SWLS

	Coeff	BootMean	BootSE	BootLLCI	BootULCI
constant	21.966	21.966	.644	20.695	23.194
X1	-4.657	-4.704	.934	-6.540	-2.845
X2	-4.674	-4.720	1.213	-7.075	-2.264
FTAS	3.906	3.943	2.202	-.458	8.089
Int_1	3.465	3.772	5.669	-7.030	15.012
Int_2	-18.843	-18.789	6.227	-31.204	-6.886

********************** ANALYSIS NOTES AND ERRORS ************************

Level of confidence for all confidence intervals in output: 95.0000

Number of bootstrap samples for percentile bootstrap confidence intervals: 10000

W values in conditional tables are the mean and +/- SD from the mean.

NOTE: The contrast option is not available with a multicategorical X.

NOTE: The following variables were mean centered prior to analysis: FTAS

STUDY MODEL 2

Moderation effect of Negative Affectivity and Social Inhibition as two subcomponents of Type D personality on the relationship between acne severity and satisfaction with life: An additive approach

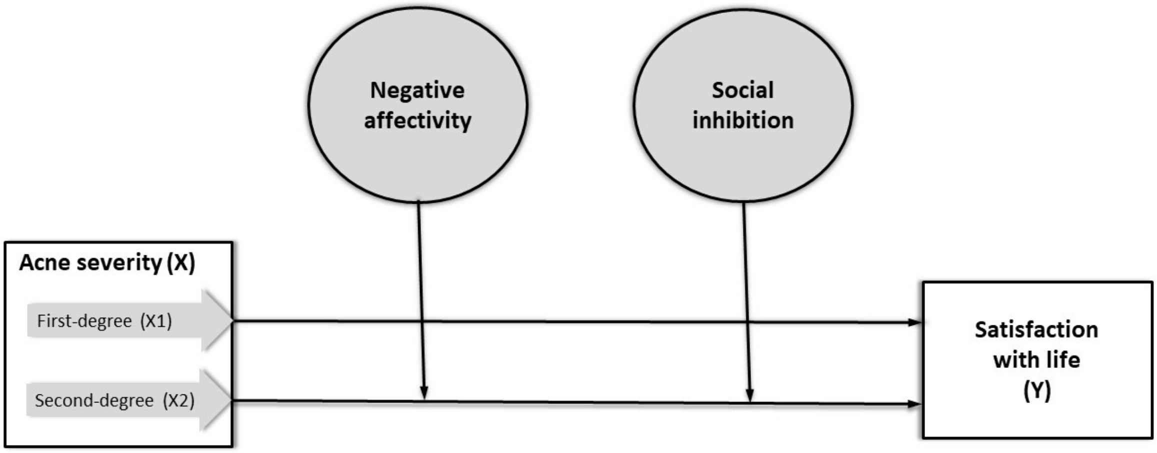

Figure S2. Study Model 2.

****************** PROCESS Procedure for SPSS Version 3.4.1 ******************
Written by Andrew F. Hayes, Ph.D. www.afhayes.com
Documentation available in Hayes (2018). www.guilford.com/p/hayes3
****

Model : 2
Y : SWLS
X : HVS
W : DS14_NE
Z : DS14_SI
Sample Size: 300
Coding of categorical X variable for analysis:

HVS	X1	X2
.000	.000	.000
1.000	1.000	.000
2.000	.000	1.000

****

OUTCOME VARIABLE: SWLS
Model Summary

R	R-sq	MSE	F	df1	df2	p
.468	.219	37.736	10.181	8.000	291.000	.000

Model

	coeff	se	t	p	LLCI	ULCI
constant	20.616	.570	36.185	.000	19.494	21.737
X1	-.902	.932	-.968	.334	-2.736	.931
X2	-4.067	1.272	-3.198	.002	-6.570	-1.564
DS14_NE	-.196	.088	-2.241	.026	-.368	-.024
Int_1	-.068	.171	-.398	.691	-.404	.268
Int_2	.194	.311	.622	.535	-.419	.807
DS14_SI	-.148	.100	-1.477	.141	-.345	.049
Int_3	-.350	.177	-1.974	.049	-.699	-.001
Int_4	-.110	.261	-.422	.673	-.625	.404

Product terms key:

Int_1	:	X1	x	DS14_NE
Int_2	:	X2	x	DS14_NE
Int_3	:	X1	x	DS14_SI
Int_4	:	X2	x	DS14_SI

Covariance matrix of regression parameter estimates:

	constant	X1	X2	DS14_NE	Int_1	Int_2	DS14_SI	Int_3	Int_4
constant	.325	-.325	-.325	.012	-.012	-.012	.015	-.015	-.015
X1	-.325	.868	.325	-.012	-.026	.012	-.015	-.011	.015
X2	-.325	.325	1.617	-.012	.012	-.124	-.015	.015	-.016
DS14_NE	.012	-.012	-.012	.008	-.008	-.008	-.004	.004	.004
Int_1	-.012	-.026	.012	-.008	.029	.008	.004	-.012	-.004
Int_2	-.012	.012	-.124	-.008	.008	.097	.004	-.004	-.054
DS14_SI	.015	-.015	-.015	-.004	.004	.004	.010	-.010	-.010
Int_3	-.015	-.011	.015	.004	-.012	-.004	-.010	.031	.010
Int_4	-.015	.015	-.016	.004	-.004	-.054	-.010	.010	.068

Test(s) of highest order unconditional interaction(s):

	R2-chng	F	df1	df2	p
X*W	.002	.315	2.000	291.000	.730
X*Z	.010	1.949	2.000	291.000	.144
BOTH	.017	1.556	4.000	291.000	.186

****

*********** BOOTSTRAP RESULTS FOR REGRESSION MODEL PARAMETERS ************

OUTCOME VARIABLE: SWLS

	Coeff	BootMean	BootSE	BootLLCI	BootULCI
constant	20.616	20.611	.677	19.285	21.944
X1	-.902	-.972	.932	-2.836	.822
X2	-4.067	-3.989	1.419	-6.595	-1.107
DS14_NE	-.196	-.195	.106	-.398	.022
Int_1	-.068	-.055	.188	-.407	.330
Int_2	.194	.174	.358	-.552	.870
DS14_SI	-.148	-.149	.111	-.374	.063
Int_3	-.350	-.347	.169	-.683	-.022
Int_4	-.110	-.093	.244	-.569	.400

********************** ANALYSIS NOTES AND ERRORS ***********************

Level of confidence for all confidence intervals in output: 95.0000

Number of bootstrap samples for percentile bootstrap confidence intervals: 10000

NOTE: The contrast option is not available with a multicategorical X.

NOTE: The following variables were mean centered prior to analysis: DS14_NE DS14_SI

STUDY MODEL 3

Moderation effect of Negative Affectivity and Social Inhibition as two subcomponents of Type D personality on the relationship between acne severity and satisfaction with life: A synergistic approach

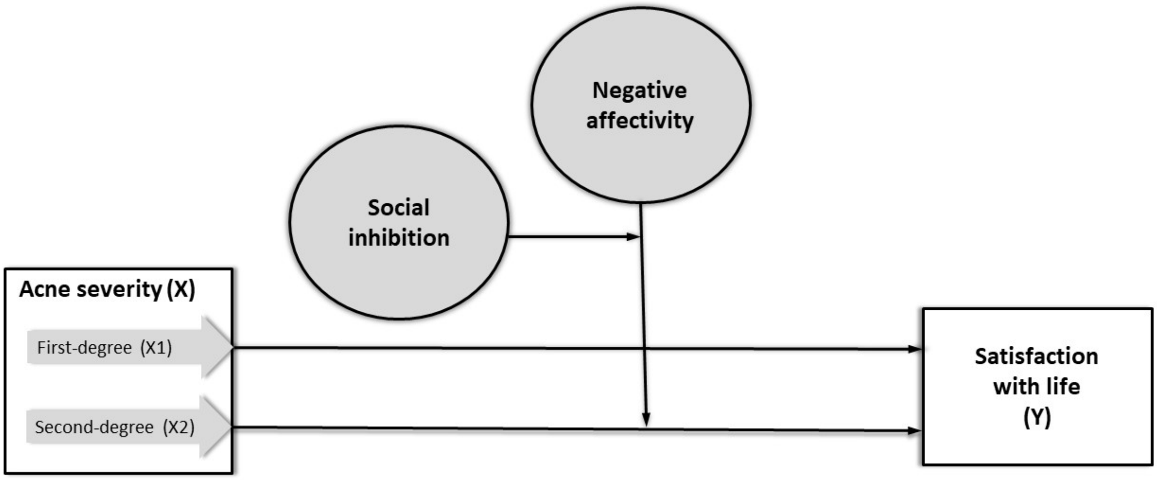

Figure S3. Study Model 3.

**************** PROCESS Procedure for SPSS Version 3.4.1 ****************

Written by Andrew F. Hayes, Ph.D. www.afhayes.com

Documentation available in Hayes (2018). www.guilford.com/p/hayes3

****

Model : 3
Y : SWLS
X : HVS
W : DS14_NE
Z : DS14_SI
Sample Size: 300
Coding of categorical X variable for analysis:

HVS	X1	X2
.000	.000	.000
1.000	1.000	.000
2.000	.000	1.000

****

OUTCOME VARIABLE: SWLS

Model Summary

R	R-sq	MSE	F	df1	df2	p
.475	.225	37.803	7.617	11.000	288.000	.000

Model

	coeff	se	t	p	LLCI	ULCI
constant	20.608	.623	33.059	.000	19.381	21.835
X1	-1.158	.987	-1.173	.242	-3.100	.784
X2	-3.791	1.333	-2.844	.005	-6.414	-1.167
DS14_NE	-.195	.095	-2.049	.041	-.382	-.008
Int_1	-.071	.175	-.408	.684	-.415	.273

Int_2	.267	.325	.822	.412	-.373	.908
DS14_SI	-.148	.101	-1.475	.141	-.346	.050
Int_3	-.409	.183	-2.231	.026	-.769	-.048
Int_4	.034	.309	.110	.912	-.574	.642
Int_5	.000	.014	.032	.974	-.027	.028
Int_6	.030	.027	1.103	.271	-.024	.084
Int_7	-.041	.048	-.849	.396	-.136	.054

Product terms key:

Int_1 : X1 x DS14_NE
Int_2 : X2 x DS14_NE
Int_3 : X1 x DS14_SI
Int_4 : X2 x DS14_SI
Int_5 : DS14_NE x DS14_SI
Int_6 : X1 x DS14_NE x DS14_SI
Int_7 : X2 x DS14_NE x DS14_SI

Covariance matrix of regression parameter estimates:

	constant	X1	X2	DS14_NE	Int_1	Int_2	DS14_SI	Int_3	Int_4	Int_5	Int_6
constant	.389	-.389	-.389	.002	-.002	-.002	.017	-.017	-.017	-.004	.004
X1	-.389	.973	.389	-.002	-.036	.002	-.017	.000	.017	.004	-.008
X2	-.389	.389	1.777	-.002	.002	-.108	-.017	.017	.036	.004	-.004
DS14_NE	.002	-.002	-.002	.009	-.009	-.009	-.004	.004	.004	.001	-.001
Int_1	-.002	-.036	.002	-.009	.031	.009	.004	-.012	-.004	-.001	.000
Int_2	-.002	.002	-.108	-.009	.009	.106	.004	-.004	-.041	-.001	.001
DS14_SI	.017	-.017	-.017	-.004	.004	.004	.010	-.010	-.010	.000	.000
Int_3	-.017	.000	.017	.004	-.012	-.004	-.010	.034	.010	.000	-.001
Int_4	-.017	.017	.036	.004	-.004	-.041	-.010	.010	.095	.000	.000
Int_5	-.004	.004	.004	.001	-.001	-.001	.000	.000	.000	.000	.000
Int_6	.004	-.008	-.004	-.001	.000	.001	.000	-.001	.000	.000	.001
Int_7	.004	-.004	-.018	-.001	.001	-.003	.000	.000	-.008	.000	.000

Test(s) of highest order unconditional interaction(s):

	R2-chng	F	df1	df2	p
X*W*Z	.006	1.137	2.000	288.000	.322

****
*********** BOOTSTRAP RESULTS FOR REGRESSION MODEL PARAMETERS ************

OUTCOME VARIABLE: SWLS

	Coeff	BootMean	BootSE	BootLLCI	BootULCI
constant	20.608	20.598	.733	19.141	22.024
X1	-1.158	-1.229	.970	-3.147	.670
X2	-3.791	-3.779	1.419	-6.470	-.893
DS14_NE	-.195	-.188	.122	-.416	.069
Int_1	-.071	-.058	.201	-.432	.354
Int_2	.267	.242	.396	-.568	.994
DS14_SI	-.148	-.153	.114	-.382	.067
Int_3	-.409	-.399	.173	-.735	-.056
Int_4	.034	.052	.269	-.472	.596
Int_5	.000	.001	.018	-.034	.038
Int_6	.030	.027	.027	-.032	.076
Int_7	-.041	-.039	.044	-.129	.049

********************** ANALYSIS NOTES AND ERRORS **********************
Level of confidence for all confidence intervals in output: 95.0000
Number of bootstrap samples for percentile bootstrap confidence intervals: 10000
NOTE: The contrast option is not available with a multicategorical X.
NOTE: The following variables were mean centered prior to analysis: DS14_NE DS14_SI

STUDY MODEL 4

Mediation effect of Negative Affectivity and Social Inhibition as two subcomponents of Type D personality on the relationship between acne severity and satisfaction with life: An additive approach

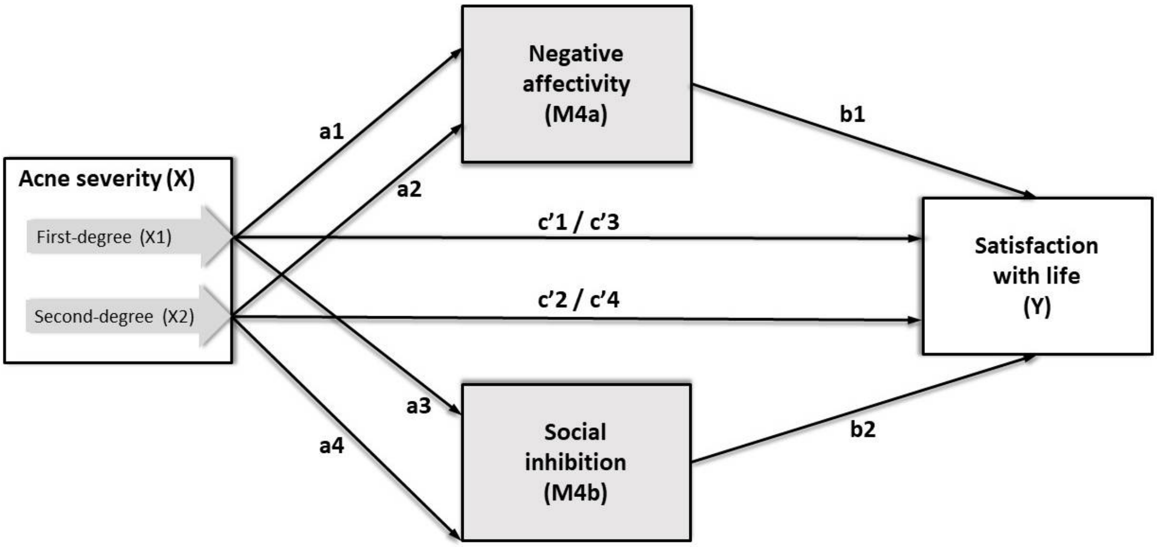

Figure S4. Study Model 4.

STUDY MODEL 4a

Negative Affectivity as a mediator

***************** PROCESS Procedure for SPSS Version 3.4.1 *****************
Written by Andrew F. Hayes, Ph.D. www.afhayes.com
Documentation available in Hayes (2018). www.guilford.com/p/hayes3
****

Model : 4
Y : SWLS
X : HVS
M : DS14_NE
Sample Size: 300
Coding of categorical X variable for analysis:

HVS	X1	X2
.000	.000	.000
1.000	1.000	.000
2.000	.000	1.000

****

OUTCOME VARIABLE: DS14_NE

Model Summary

R	R-sq	MSE	F	df1	df2	p
.468	.219	30.469	41.711	2.000	297.000	.000

Model

	coeff	se	t	p	LLCI	ULCI
constant	11.880	.451	26.359	.000	10.993	12.767
X1	5.412	.721	7.501	.000	3.992	6.832
X2	6.435	.876	7.346	.000	4.711	8.159

Standardized coefficients

	coeff
X1	.869
X2	1.033

Covariance matrix of regression parameter estimates:

	constant	X1	X2
constant	.203	-.203	-.203
X1	-.203	.521	.203
X2	-.203	.203	.767

****

OUTCOME VARIABLE: SWLS

Model Summary

R	R-sq	MSE	F	df1	df2	p
.418	.175	39.195	20.864	3.000	296.000	.000

Model

	coeff	se	t	p	LLCI	ULCI
constant	25.065	.934	26.833	.000	23.227	26.904
X1	-1.983	.892	-2.222	.027	-3.739	-.226
X2	-4.071	1.080	-3.769	.000	-6.196	-1.945
DS14_NE	-.294	.066	-4.467	.000	-.423	-.164

Standardized coefficients

	coeff
X1	-.289
X2	-.594
DS14_NE	-.267

Covariance matrix of regression parameter estimates:

	constant	X1	X2	DS14_NE
constant	.873	.017	.070	-.051
X1	.017	.796	.412	-.023
X2	.070	.412	1.166	-.028
DS14_NE	-.051	-.023	-.028	.004

Test(s) of X by M interaction:

F	df1	df2	p
.730	2.000	294.000	.483

************************* TOTAL EFFECT MODEL **************************
OUTCOME VARIABLE: SWLS

Model Summary

R	R-sq	MSE	F	df1	df2	p
.345	.119	41.695	20.042	2.000	297.000	.000

Model

	coeff	se	t	p	LLCI	ULCI
constant	21.573	.527	40.918	.000	20.536	22.611
X1	-3.573	.844	-4.234	.000	-5.234	-1.912
X2	-5.962	1.025	-5.818	.000	-7.979	-3.946

Standardized coefficients

	coeff
X1	-.521
X2	-.870

Covariance matrix of regression parameter estimates:

	constant	X1	X2
constant	.278	-.278	-.278
X1	-.278	.712	.278
X2	-.278	.278	1.050

************* TOTAL, DIRECT, AND INDIRECT EFFECTS OF X ON Y *************

Relative total effects of X on Y:

	Effect	se	t	p	LLCI	ULCI	c_ps
X1	-3.573	.844	-4.234	.000	-5.234	-1.912	-.521
X2	-5.962	1.025	-5.818	.000	-7.979	-3.946	-.870

Omnibus test of total effect of X on Y:

R2-chng	F	df1	df2	p
.119	20.042	2.000	297.000	.000

Relative direct effects of X on Y

	Effect	se	t	p	LLCI	ULCI	c'_ps
X1	-1.983	.892	-2.222	.027	-3.739	-.226	-.289
X2	-4.071	1.080	-3.769	.000	-6.196	-1.945	-.594

Omnibus test of direct effect of X on Y:

R2-chng	F	df1	df2	p
.041	7.331	2.000	296.000	.001

Relative indirect effects of X on Y: HVS -> DS14_NE -> SWLS

	Effect	BootSE	BootLLCI	BootULCI
X1	-1.591	.435	-2.447	-.724
X2	-1.892	.551	-2.993	-.821

Partially standardized relative indirect effect(s) of X on Y: HVS -> DS14_NE -> SWLS

	Effect	BootSE	BootLLCI	BootULCI
X1	-.232	.062	-.352	-.108
X2	-.276	.078	-.430	-.121

****

*********** BOOTSTRAP RESULTS FOR REGRESSION MODEL PARAMETERS ************

OUTCOME VARIABLE: DS14_NE

	Coeff	BootMean	BootSE	BootLLCI	BootULCI
constant	11.880	11.881	.531	10.831	12.911
X1	5.412	5.407	.706	3.997	6.761
X2	6.435	6.436	.743	4.972	7.890

OUTCOME VARIABLE: SWLS

	Coeff	BootMean	BootSE	BootLLCI	BootULCI
constant	25.065	25.053	1.125	22.771	27.271
X1	-1.983	-2.018	.906	-3.827	-.273
X2	-4.071	-4.087	1.078	-6.188	-1.956
DS14_NE	-.294	-.292	.080	-.448	-.131

*********************** ANALYSIS NOTES AND ERRORS ************************

Level of confidence for all confidence intervals in output: 95.0000
Number of bootstrap samples for percentile bootstrap confidence intervals: 10000
NOTE: Standardized coefficients for dichotomous or multicategorical X are in partially standardized form.

STUDY MODEL 4b

Social Inhibition as a mediator

****************** PROCESS Procedure for SPSS Version 3.4.1 ******************
Written by Andrew F. Hayes, Ph.D. www.afhayes.com
Documentation available in Hayes (2018). www.guilford.com/p/hayes3

****

Model : 4
Y : SWLS
X : HVS
M : DS14_SI
Sample Size: 300
Coding of categorical X variable for analysis:

HVS	X1	X2
.000	.000	.000
1.000	1.000	.000
2.000	.000	1.000

****

OUTCOME VARIABLE: DS14_SI

Model Summary

R	R-sq	MSE	F	df1	df2	p
.461	.213	27.010	40.128	2.000	297.000	.000

Model

	coeff	se	t	p	LLCI	ULCI
constant	8.507	.424	20.047	.000	7.672	9.342
X1	4.743	.679	6.983	.000	3.407	6.080
X2	6.234	.825	7.559	.000	4.611	7.857

Standardized coefficients

	coeff
X1	.813
X2	1.068

Covariance matrix of regression parameter estimates:

	constant	X1	X2
constant	.180	-.180	-.180
X1	-.180	.461	.180
X2	-.180	.180	.680

****

OUTCOME VARIABLE: SWLS

Model Summary

R	R-sq	MSE	F	df1	df2	p
.429	.184	38.743	22.256	3.000	296.000	.000

Model

	coeff	se	t	p	LLCI	ULCI
constant	24.447	.780	31.358	.000	22.913	25.981
X1	-1.971	.878	-2.245	.025	-3.698	-.243
X2	-3.856	1.079	-3.575	.000	-5.979	-1.733
DS14_SI	-.338	.069	-4.861	.000	-.475	-.201

Standardized coefficients

	coeff
X1	-.287
X2	-.562
DS14_SI	-.288

Covariance matrix of regression parameter estimates:

	constant	X1	X2	DS14_SI
constant	.608	-.063	-.002	-.041
X1	-.063	.771	.401	-.023
X2	-.002	.401	1.163	-.030
DS14_SI	-.041	-.023	-.030	.005

Test(s) of X by M interaction:

F	df1	df2	p
2.273	2.000	294.000	.105

************************ TOTAL EFFECT MODEL **************************

OUTCOME VARIABLE: SWLS

Model Summary

R	R-sq	MSE	F	df1	df2	p
.345	.119	41.695	20.042	2.000	297.000	.000

Model

	Coeff	se	t	p	LLCI	ULCI
constant	21.573	.527	40.918	.000	20.536	22.611
X1	-3.573	.844	-4.234	.000	-5.234	-1.912
X2	-5.962	1.025	-5.818	.000	-7.979	-3.946

Standardized coefficients

	coeff
X1	-.521
X2	-.870

Covariance matrix of regression parameter estimates:

	constant	X1	X2
constant	.278	-.278	-.278
X1	-.278	.712	.278
X2	-.278	.278	1.050

************** TOTAL, DIRECT, AND INDIRECT EFFECTS OF X ON Y **************

Relative total effects of X on Y:

	Effect	se	t	p	LLCI	ULCI	c_ps
X1	-3.573	.844	-4.234	.000	-5.234	-1.912	-.521
X2	-5.962	1.025	-5.818	.000	-7.979	-3.946	-.870

Omnibus test of total effect of X on Y:

R2-chng	F	df1	df2	p
.119	20.042	2.000	297.000	.000

Relative direct effects of X on Y

	Effect	se	t	p	LLCI	ULCI	c'_ps
X1	-1.971	.878	-2.245	.025	-3.698	-.243	-.287
X2	-3.856	1.079	-3.575	.000	-5.979	-1.733	-.562

Omnibus test of direct effect of X on Y:

R2-chng	F	df1	df2	p
.037	6.716	2.000	296.000	.001

Relative indirect effects of X on Y: HVS -> DS14_SI -> SWLS

	Effect	BootSE	BootLLCI	BootULCI
X1	-1.602	.431	-2.541	-.834
X2	-2.106	.585	-3.367	-1.079

Partially standardized relative indirect effect(s) of X on Y: HVS -> DS14_SI -> SWLS

	Effect	BootSE	BootLLCI	BootULCI
X1	-.234	.061	-.366	-.125
X2	-.307	.082	-.482	-.160

****

*********** BOOTSTRAP RESULTS FOR REGRESSION MODEL PARAMETERS ************

OUTCOME VARIABLE: DS14_SI

	Coeff	BootMean	BootSE	BootLLCI	BootULCI
constant	8.507	8.503	.470	7.586	9.429
X1	4.743	4.750	.662	3.440	6.058
X2	6.234	6.232	.815	4.619	7.811

OUTCOME VARIABLE: SWLS

	Coeff	BootMean	BootSE	BootLLCI	BootULCI
constant	24.447	24.447	.810	22.815	26.018
X1	-1.971	-1.975	.905	-3.733	-.183
X2	-3.856	-3.838	1.083	-5.929	-1.636
DS14_SI	-.338	-.338	.075	-.484	-.193

********************** ANALYSIS NOTES AND ERRORS ************************
Level of confidence for all confidence intervals in output: 95.0000
Number of bootstrap samples for percentile bootstrap confidence intervals: 10000
NOTE: Standardized coefficients for dichotomous or multicategorical X are in partially standardized form.

STUDY MODEL 5

Mediation effect of Negative Affectivity and Social Inhibition as two subcomponents of Type D personality on the relationship between acne severity and satisfaction with life: An additive approach

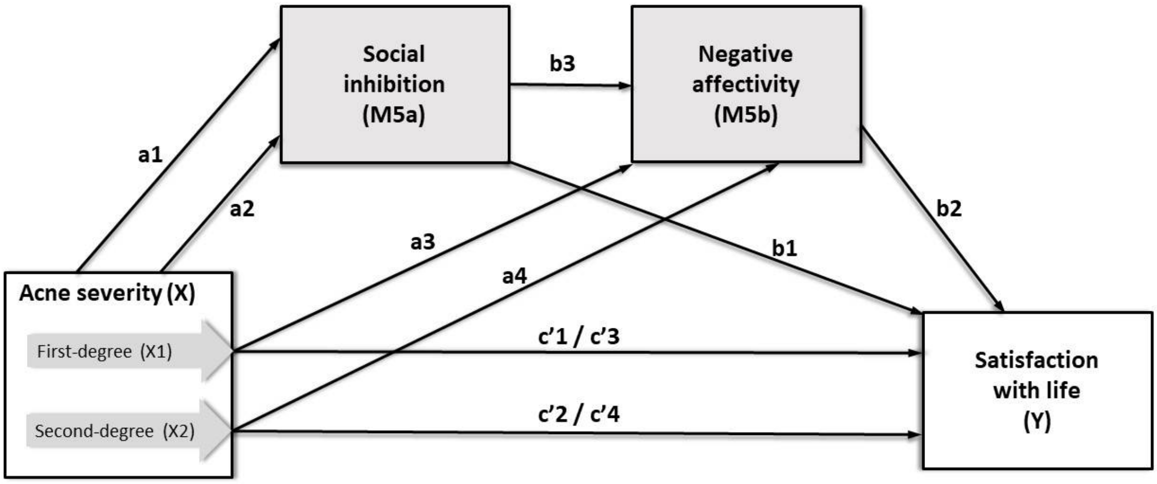

Figure S5. Study Model 5.

**************** PROCESS Procedure for SPSS Version 3.4.1 ****************
Written by Andrew F. Hayes, Ph.D. www.afhayes.com
Documentation available in Hayes (2018). www.guilford.com/p/hayes3
****

Model : 6
Y : SWLS
X : HVS
M1 : DS14_SI
M2 : DS14_NE
Sample Size: 300
Coding of categorical X variable for analysis:

HVS	X1	X2
.000	.000	.000
1.000	1.000	.000
2.000	.000	1.000

****

OUTCOME VARIABLE: DS14_SI

Model Summary

R	R-sq	MSE	F	df1	df2	p
.461	.213	27.010	40.128	2.000	297.000	.000

Model

	coeff	se	t	p	LLCI	ULCI
constant	8.507	.424	20.047	.000	7.672	9.342
X1	4.743	.679	6.983	.000	3.407	6.080
X2	6.234	.825	7.559	.000	4.611	7.857

Standardized coefficients

	coeff
X1	.813
X2	1.068

Covariance matrix of regression parameter estimates:

	constant	X1	X2
constant	.180	-.180	-.180
X1	-.180	.461	.180
X2	-.180	.180	.680

****

OUTCOME VARIABLE: DS14_NE

Model Summary

R	R-sq	MSE	F	df1	df2	p
.619	.383	24.165	61.224	3.000	296.000	.000

Model

	coeff	se	t	p	LLCI	ULCI
constant	7.744	.616	12.577	.000	6.532	8.955
X1	3.105	.693	4.479	.000	1.741	4.470
X2	3.404	.852	3.995	.000	1.727	5.080
DS14_SI	.486	.055	8.859	.000	.378	.594

Standardized coefficients

	coeff
X1	.499
X2	.547
DS14_SI	.456

Covariance matrix of regression parameter estimates:

	constant	X1	X2	DS14_SI
constant	.379	-.040	-.001	-.026
X1	-.040	.481	.250	-.014
X2	-.001	.250	.726	-.019
DS14_SI	-.026	-.014	-.019	.003

Test(s) of X by M interaction:

	F	df1	df2	p
M1*X	.984	2.000	294.000	.375

****

OUTCOME VARIABLE: SWLS

Model Summary

R	R-sq	MSE	F	df1	df2	p
.449	.202	38.021	18.666	4.000	295.000	.000

Model

	coeff	se	t	p	LLCI	ULCI
constant	25.900	.957	27.074	.000	24.018	27.783
X1	-1.388	.899	-1.545	.123	-3.157	.380
X2	-3.217	1.097	-2.933	.004	-5.376	-1.059
DS14_SI	-.247	.077	-3.184	.002	-.399	-.094
DS14_NE	-.188	.073	-2.574	.011	-.331	-.044

Standardized coefficients

	coeff
X1	-.202
X2	-.469
DS14_SI	-.210
DS14_NE	-.170

Covariance matrix of regression parameter estimates:

	constant	X1	X2	DS14_SI	DS14_NE
constant	.915	.066	.138	-.020	-.041
X1	.066	.807	.450	-.014	-.017
X2	.138	.450	1.203	-.021	-.018
DS14_SI	-.020	-.014	-.021	.006	-.003
DS14_NE	-.041	-.017	-.018	-.003	.005

Test(s) of X by M interaction:

	F	df1	df2	p
M1*X	2.810	2.000	293.000	.062
M2*X	1.155	2.000	293.000	.317

************************** TOTAL EFFECT MODEL ****************************

OUTCOME VARIABLE: SWLS

Model Summary

R	R-sq	MSE	F	df1	df2	p
.345	.119	41.695	20.042	2.000	297.000	.000

Model

	coeff	se	t	p	LLCI	ULCI
constant	21.573	.527	40.918	.000	20.536	22.611
X1	-3.573	.844	-4.234	.000	-5.234	-1.912
X2	-5.962	1.025	-5.818	.000	-7.979	-3.946

Standardized coefficients

	coeff
X1	-.521
X2	-.870

Covariance matrix of regression parameter estimates:

	constant	X1	X2
constant	.278	-.278	-.278
X1	-.278	.712	.278
X2	-.278	.278	1.050

************** TOTAL, DIRECT, AND INDIRECT EFFECTS OF X ON Y **************

Relative total effects of X on Y:

	Effect	se	t	p	LLCI	ULCI	c_ps
X1	-3.573	.844	-4.234	.000	-5.234	-1.912	-.521
X2	-5.962	1.025	-5.818	.000	-7.979	-3.946	-.870

Omnibus test of total effect of X on Y:

R2-chng	F	df1	df2	p
.119	20.042	2.000	297.000	.000

Relative direct effects of X on Y

	Effect	se	t	p	LLCI	ULCI	c'_ps
X1	-1.388	.899	-1.545	.123	-3.157	.380	-.202
X2	-3.217	1.097	-2.933	.004	-5.376	-1.059	-.469

Omnibus test of direct effect of X on Y:

R2-chng	F	df1	df2	p
.023	4.328	2.000	295.000	.014

Relative indirect effects of X on Y:

HVS -> DS14_SI -> SWLS

	Effect	BootSE	BootLLCI	BootULCI
X1	-1.170	.433	-2.114	-.413
X2	-1.537	.572	-2.779	-.540

HVS -> DS14_NE -> SWLS

	Effect	BootSE	BootLLCI	BootULCI
X1	-.583	.271	-1.137	-.055
X2	-.639	.309	-1.275	-.056

HVS -> DS14_SI -> DS14_NE -> SWLS

	Effect	BootSE	BootLLCI	BootULCI
X1	-.433	.208	-.869	-.037
X2	-.569	.281	-1.145	-.046

Partially standardized relative indirect effect(s) of X on Y:

HVS -> DS14_SI -> SWLS

	Effect	BootSE	BootLLCI	BootULCI
X1	-.171	.062	-.305	-.061
X2	-.224	.082	-.401	-.080

HVS -> DS14_NE -> SWLS

	Effect	BootSE	BootLLCI	BootULCI
X1	-.085	.040	-.165	-.008

	Effect	BootSE	BootLLCI	BootULCI
X2	-.093	.045	-.187	-.008

HVS -> DS14_SI -> DS14_NE -> SWLS

	Effect	BootSE	BootLLCI	BootULCI
X1	-.063	.030	-.125	-.005
X2	-.083	.041	-.166	-.007

****

*********** BOOTSTRAP RESULTS FOR REGRESSION MODEL PARAMETERS ************

OUTCOME VARIABLE: DS14_SI

	Coeff	BootMean	BootSE	BootLLCI	BootULCI
constant	8.507	8.512	.459	7.604	9.408
X1	4.743	4.743	.661	3.419	6.007
X2	6.234	6.236	.810	4.639	7.811

OUTCOME VARIABLE: DS14_NE

	Coeff	BootMean	BootSE	BootLLCI	BootULCI
constant	7.744	7.746	.712	6.337	9.166
X1	3.105	3.111	.693	1.745	4.477
X2	3.404	3.409	.682	2.091	4.784
DS14_SI	.486	.486	.057	.374	.597

OUTCOME VARIABLE: SWLS

	Coeff	BootMean	BootSE	BootLLCI	BootULCI
constant	25.900	25.890	1.117	23.692	28.054
X1	-1.388	-1.383	.937	-3.242	.439
X2	-3.217	-3.198	1.146	-5.426	-.950
DS14_SI	-.247	-.247	.078	-.403	-.095
DS14_NE	-.188	-.187	.085	-.351	-.016

********************** ANALYSIS NOTES AND ERRORS ***********************

Level of confidence for all confidence intervals in output: 95.0000

Number of bootstrap samples for percentile bootstrap confidence intervals: 10000

NOTE: Standardized coefficients for dichotomous or multicategorical X are in partially standardized form.

STUDY MODEL 6

Moderation mediation effect of interaction between Negative Affectivity and Social Inhibition on the relationship between acne severity and satisfaction with life: A synergistic approach

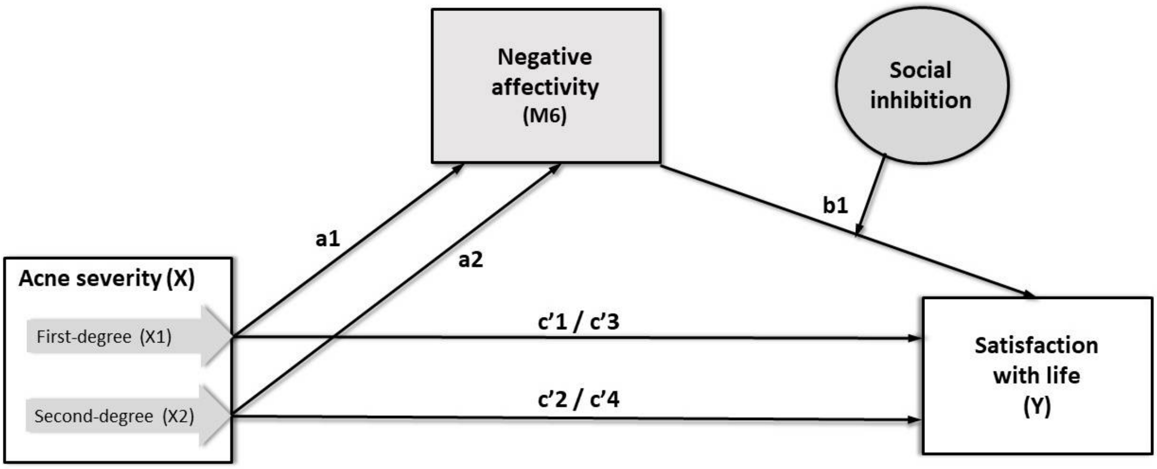

Figure S6. Study Model 6.

****************** PROCESS Procedure for SPSS Version 3.4.1 ******************
Written by Andrew F. Hayes, Ph.D. www.afhayes.com
Documentation available in Hayes (2018). www.guilford.com/p/hayes3
****

Model : 14
Y : SWLS
X : HVS
M : DS14_NE
W : DS14_SI
Sample Size: 300
Coding of categorical X variable for analysis:

HVS	X1	X2
.000	.000	.000
1.000	1.000	.000
2.000	.000	1.000

****

OUTCOME VARIABLE: DS14_NE

Model Summary

R	R-sq	MSE	F	df1	df2	p
.468	.219	30.469	41.711	2.000	297.000	.000

Model

	coeff	se	t	p	LLCI	ULCI
constant	-2.890	.451	-6.412	.000	-3.777	-2.003
X1	5.412	.721	7.501	.000	3.992	6.832
X2	6.435	.876	7.346	.000	4.711	8.159

Covariance matrix of regression parameter estimates:

	constant	X1	X2
constant	.203	-.203	-.203
X1	-.203	.521	.203
X2	-.203	.203	.767

****

OUTCOME VARIABLE: SWLS

Model Summary

R	R-sq	MSE	F	df1	df2	p
.450	.202	38.144	14.894	5.000	294.000	.000

Model

	coeff	se	t	p	LLCI	ULCI
constant	20.326	.604	33.669	.000	19.138	21.514
X1	-1.367	.906	-1.509	.132	-3.149	.416
X2	-3.225	1.099	-2.934	.004	-5.389	-1.062
DS14_NE	-.184	.075	-2.438	.015	-.332	-.035
DS14_SI	-.250	.079	-3.156	.002	-.406	-.094
Int_1	.002	.011	.216	.829	-.019	.024

Product terms key:

Int_1 : DS14_NE x DS14_SI

Covariance matrix of regression parameter estimates:

	constant	X1	X2	DS14_NE	DS14_SI	Int_1
constant	.364	-.366	-.352	.004	.012	-.003
X1	-.366	.820	.448	-.015	-.016	.001
X2	-.352	.448	1.209	-.019	-.020	.000
DS14_NE	.004	-.015	-.019	.006	-.003	.000
DS14_SI	.012	-.016	-.020	-.003	.006	.000
Int_1	-.003	.001	.000	.000	.000	.000

Test(s) of X by M interaction:

F	df1	df2	p
1.163	2.000	292.000	.314

Test(s) of highest order unconditional interaction(s):

	R2-chng	F	df1	df2	p
M*W	.000	.047	1.000	294.000	.829

***************** DIRECT AND INDIRECT EFFECTS OF X ON Y ****************

Relative direct effects of X on Y

	Effect	se	t	p	LLCI	ULCI
X1	-1.367	.906	-1.509	.132	-3.149	.416
X2	-3.225	1.099	-2.934	.004	-5.389	-1.062

Omnibus test of direct effect of X on Y:

R2-chng	F	df1	df2	p
.023	4.326	2.000	294.000	.014

Relative conditional indirect effects of X on Y:

INDIRECT EFFECT: HVS -> DS14_NE -> SWLS

	DS14_SI	Effect	BootSE	BootLLCI	BootULCI
X1	-5.838	-1.068	.549	-2.092	.089
X1	.000	-.994	.463	-1.857	-.032
X1	5.838	-.920	.667	-2.196	.458

Index of moderated mediation:

	Index	BootSE	BootLLCI	BootULCI
DS14_SI	.013	.068	-.127	.144

	DS14_SI	Effect	BootSE	BootLLCI	BootULCI
X2	-5.838	-1.270	.676	-2.574	.097
X2	.000	-1.182	.570	-2.288	-.039
X2	5.838	-1.094	.799	-2.634	.526

Index of moderated mediation:

	Index	BootSE	BootLLCI	BootULCI
DS14_SI	.015	.081	-.147	.173

****

*********** BOOTSTRAP RESULTS FOR REGRESSION MODEL PARAMETERS ************

OUTCOME VARIABLE: DS14_NE

	Coeff	BootMean	BootSE	BootLLCI	BootULCI
constant	-2.890	-2.889	.518	-3.916	-1.891
X1	5.412	5.414	.703	4.011	6.776
X2	6.435	6.424	.747	4.979	7.884

OUTCOME VARIABLE: SWLS

	Coeff	BootMean	BootSE	BootLLCI	BootULCI
constant	20.326	20.322	.719	18.904	21.729
X1	-1.367	-1.386	.954	-3.217	.462
X2	-3.225	-3.221	1.137	-5.421	-.973
DS14_NE	-.184	-.180	.086	-.344	-.006
DS14_SI	-.250	-.252	.083	-.416	-.090
Int_1	.002	.003	.013	-.023	.027

********************** ANALYSIS NOTES AND ERRORS ***********************

Level of confidence for all confidence intervals in output: 95.0000

Number of bootstrap samples for percentile bootstrap confidence intervals: 10000

W values in conditional tables are the mean and +/- SD from the mean.

NOTE: The following variables were mean centered prior to analysis: DS14_SI, DS14_NE

NOTE: Standardized coefficients not available for models with moderators.
